# The efficacy of intramuscular ephedrine in preventing hemodynamic perturbations in patients with spinal anesthesia and dexmedetomidine sedation

**DOI:** 10.7150/ijms.48772

**Published:** 2020-08-25

**Authors:** Ji-Hyoung Park, Jae-Kwang Shim, Hyejin Hong, Hyun Kyo Lim

**Affiliations:** 1 Department of Anesthesiology and Pain Medicine, Yonsei University Wonju College of Medicine, Wonju, Gangwon-do, Republic of Korea; 2 Department of Anesthesiology and Pain Medicine, and Anesthesia and Pain Research Institute, Yonsei University College of Medicine, Seoul, Republic of Korea

**Keywords:** Bradycardia, Dexmedetomidine, Ephedrine, Hypotension

## Abstract

Dexmedetomidine is used for sedation during spinal anesthesia. The sympatholytic effect of dexmedetomidine may exacerbate hypotension and bradycardia with spinal anesthesia. This study investigated the effects of prophylactic intramuscular injection of ephedrine in preventing hypotension and bradycardia occurring through combined use of spinal anesthesia and dexmedetomidine. One hundred sixteen patients scheduled for lower extremity orthopedic surgery were randomized into two groups receiving either ephedrine 20 mg intramuscularly or equivalent amount of 0.9% NaCl, both with dexmedetomidine and spinal anesthesia. The primary endpoint was the incidence of hemodynamic perturbations (hypotension or bradycardia event). The secondary endpoint was a rescue doses of ephedrine and atropine. The incidence of hemodynamic perturbations was significantly lower in the ephedrine group compared with to the saline group (26.3% versus 55.9%, p = 0.001). The rescue doses of atropine (0.09 ± 0.21 versus 0.28 ± 0.41, p = 0.001) and ephedrine (1.04 ± 2.89 versus 2.03 ± 3.25, p = 0.007) were also significantly lower in the ephedrine group. There was no differences in number of patients with hypertensive (7.0% versus 11.9%, p = 0.375) or tachycardia (1.8% versus 3.4% p = 0.581) episodes. The use of ephedrine intramuscular injections may be a safe and efficacious option in preventing hemodynamic perturbations in patients who received spinal anesthesia and sedation using dexmedetomidine.

## Introduction

Proper sedation during spinal anesthesia has become essential in the current anesthetic practice in terms of patient satisfaction and compliance of regional anesthesia 1. However, sedation is inevitably accompanied by respiratory depression 2. To provide a safe operative milieu for patients undergoing spinal anesthesia requiring sedation, continuous infusion of dexmedetomidine has gained wide-spread popularity 3.

Dexmedetomidine, a highly selective alpha-2 adrenoceptor agonist, is a unique sedative that has the benefits of analgesic, sympatholytic, and respiratory-preserving properties 4. Intravenous administration of dexmedetomidine during spinal anesthesia also conveys advantages in terms of prolonging sensory and motor blocks 5, and reducing the amount of necessary postoperative analgesics 6, 7. However, dexmedetomidine reduces central sympathetic outflow, leading to decrease in blood pressure and heart rate (HR) 8, which may be aggravated by spinal anesthesia-induced blockage of the sympathetic nervous systems 9. Several studies have demonstrated that use of dexmedetomidine during spinal anesthesia increased the frequency of bradycardia and hypotension 10-12.

Ephedrine is a non-specific adrenergic stimulant possessing both direct and indirect effects, and has long been the drug of choice in managing spinal anesthesia-induced hemodynamic perturbations 13. However, ephedrine is not suitable for continuous infusion. Repeated intermittent intravenous bolus administration of ephedrine is a reactive treatment rather than a preventive one and inevitably results in oscillations of peak and trough drug levels 14. To overcome these potential shortcomings, the efficacy of a single intramuscular (IM) ephedrine injection to prevent spinal anesthesia-induced bradycardia or hypotension has been validated by previous studies that exhibited promising results 15. Unfortunately, evidence in that regard is lacking in patients receiving dexmedetomidine during spinal anesthesia.

The primary aim of this randomized controlled study was to investigate the efficacy of prophylactic IM ephedrine injection in preventing spinal anesthesia-induced hemodynamic perturbations (events of bradycardia or hypotension) in patients undergoing lower extremity orthopedic surgery.

## Method

### Study population

The current trial was approved by the Institutional Review Board of Yonsei University Wonju College of Medicine, Wonju, Korea, enlisted in https://cris.nih.go.kr (KCT0002833), and followed the CONsolidated Standards of Reporting Trials (CONSORT) 2010 guidelines. After acquiring informed consent from each patient, 120 patients with American Society of Anesthesiologist physical status I, II or III aged 20-80 years, who underwent lower extremity orthopedic surgery under spinal anesthesia between December 2017 and July 2019 were enrolled. Patients with a history of heart failure, recent myocardial infarction and/or stroke (within 3 months of surgery), cognitive impairment, hepatic dysfunction, or failed spinal anesthesia were excluded from the study. Patients were randomly and evenly assigned to either the IM ephedrine or control group by a computerized randomized table. Blinding of the group designation was maintained to the patients, attending anesthesiologists, and interventionists, while the studied drugs were prepared by an anesthesia nurse who was not involved in patient care or assessment.

### Anesthetic and procedural management

For premedication, patients received an IM injection of midazolam 1 mg before entering the operating room. In the operating room, ECG, non-invasive blood pressure, pulse-oximetry (SpO_2_), and respiratory rates were monitored in all patients. Before spinal anesthesia, hydration with balanced isotonic crystalloid 6 ml/kg intravenous administration was performed.

Spinal anesthesia was performed in the lateral decubitus position using a midline approach at the L3/4 or L4/5 intervertebral space. Intrathecal injection of 10 to 12 mg hyperbaric bupivacaine (Marcaine^®^, AstraZeneca AB, Södertälje, Sweden) was administered according to height, weight, age of patients and target sensory level of the blocks. The level of block was assessed by loss of pinprick sensation in the mid-clavicular line using a 26-gauge needle.

After confirmation of appropriate block height, dexmedetomidine infusion was initiated based on previous studies 16. In both groups, dexmedetomidine 1.0 μg/kg was administered for 10 minutes followed by continuous infusion of 0.2-0.7 μg/kg/h until the end of surgery. Dose adjustments were performed to target a Ramsay sedation score between 2 and 4 (1 = anxious and agitated, restless; 2 = co-operative, oriented and tranquil; 3 = response to commands only; 4 = brisk response; 5 = sluggish response to light glabellar tap or loud auditory stimuli; 6 = no response to stimulation) 17. At the beginning of dexmedetomidine loading dose infusion, patients in the IM ephedrine group received 20 mg of ephedrine into the ventrogluteal region (opposite the surgical site) in a total volume of 2 ml mixed with 0.9% saline, and patients in the control group received the equivalent amount of 0.9% saline.

The baseline point was defined as the starting point of dexmedetomidine loading. Hemodynamic data were serially recorded at 3, 5, 10, 30, 60, 90, and 120 minutes after baseline. If hypotension (mean arterial pressure (MAP) < 60 mmHg or > 30% decrease in MAP compared with the preanesthetic rate) occurred, 4-8 mg of ephedrine was administered intravenously. If bradycardia (HR < 45 bpm or > 30% decrease in HR compared with the preanesthetic rate) occurred, 0.5 mg of atropine was administered intravenously. If hypertension (systolic arterial pressure > 180 mmHg or diastolic arterial pressure > 110 mmHg) occurred, 0.5-1 mg of nicardipine hydrochloride was administered intravenously. If tachycardia (HR > 110 bpm) occurred, 5-10 mg of esmolol was given intravenously. If respiratory depression (respiratory rate < 10/min) and/ or hypoxia (SpO_2_ < 90%) occurred, patients were given a verbal awakening stimulation (calling their name) followed by a gentle squeeze at the trapezius muscle, if unresponsive. If respiratory depression persisted despite these stimulations, dexmedetomidine infusion was discontinued while preparing for further advanced airway management.

### Primary endpoint and assessment

The primary endpoint of the current study was to investigate the incidence of hemodynamic perturbations represented by bradycardia and/or hypotensive (defined as above) events between the groups.

### Secondary endpoints

The secondary endpoints of the current study were to determine the total amounts of rescue ephedrine and atropine and the incidences of hypertensive and tachycardia events, and nausea/vomiting requiring treatment between the groups.

### Statistical analysis

Sample size calculation was executed based on incidence of bradycardia. In a previous study, the incidence of bradycardia was 30% in patients with a combination of spinal anesthesia and dexmedetomidine 18. Assuming that the use of IM ephedrine can reduce the incidence by 8% or more, the estimated number of patients in each group was 58 patients at an α error of 0.05 and a power of 90%. Accounting for a dropout rate of 5%, we enrolled 60 patients in each group.

All statistical analyses were performed with SAS 9.4 and Rx64 programs. Comparative analysis of repeated-measurement variables was performed by repeated-measures analysis of variance (ANOVA) for between-group and within-group comparisons. Mauchy's sphericity test was performed. If the sphericity assumption was violated, the modified statistic tests as Greenhouse-Geisser and Huynh-Feldt were used. If there was a significant interaction with time and group, post hoc Bonferroni corrections were used to correct the type I error. Hemodynamic data that were serially assessed at 8 time points. Therefore, the p values for the hemodynamic data were considered statistically significant when p < 0.00625. Otherwise, p < 0.05 was considered statistically significant. Proportions were compared to χ^2^ test and pairwise comparisons between the groups were performed with paired t-tests. Intergroup comparisons of variables that showed normal distribution were tested using the independent t- test (mean ± standard deviation [SD]). In cases of abnormal distribution, the Mann-Whitney U test was performed (median [interquartile range]). Intergroup comparisons of categorical variables were conducted using the Chi-square test (n [%]).

## Results

**Figure 1:**
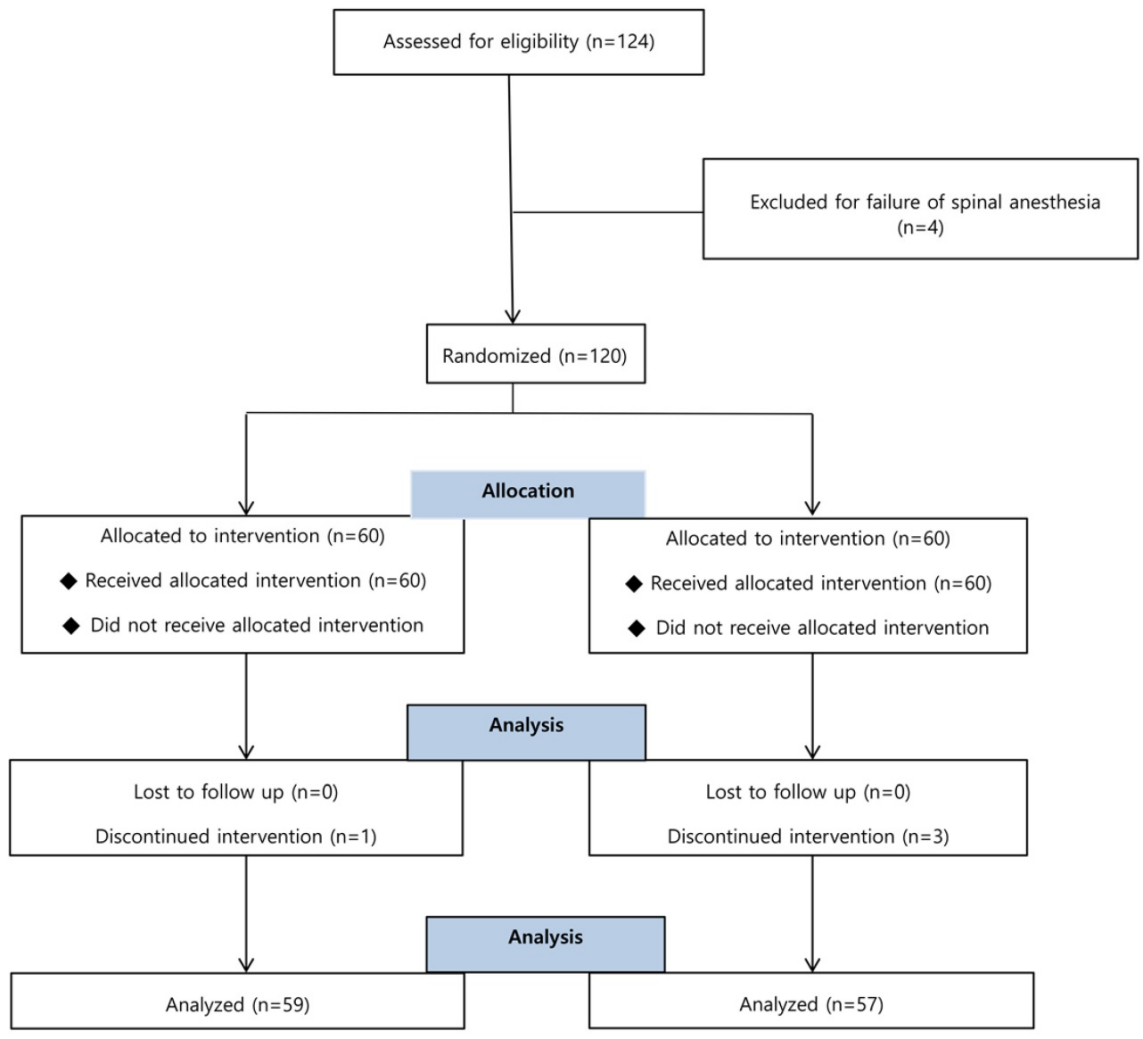
Flow chart of patient enrollment. Of the 124 patients, 4 were excluded for failure of spinal anesthesia and 4 were discontinued intervention for conversion to the general anesthesia.

A total of 124 patients was screened, and 120 of them were enrolled and randomized into either the ephedrine or control group. There were 4 dropouts among the 120 enrolled subjects due to conversion to general anesthesia (Figure 1).

Patient characteristics and procedural data are displayed in Table 1.

The incidence of hemodynamic perturbations, which was the primary endpoint, was significantly lower in the IM ephedrine group compared with the control group (55.9% versus 26.3%, p = 0.001) yielding a statistical power of 90.3% for the primary outcome variable.

The total doses of rescue atropine (0.28 ± 0.41 versus 0.09 ± 0.21, p = 0.001) and ephedrine (2.03 ± 3.25 versus 1.04 ± 2.89, p = 0.007), which were the secondary endpoint of the study were also significantly lower in the ephedrine group. The number of patients with one or more hypertensive (11.9% versus 7.0%, p = 0.375) or tachycardia (3.4% versus 1.8%, p = 0.581) episodes did not differ between the groups. The intraoperative (3.4% versus 5.3%, p = 0.621) and postoperative (69.5% versus 63.2%, p = 0.472) anti-emetic requirements were also similar between the groups (Table 2.).

**Figure 2:**
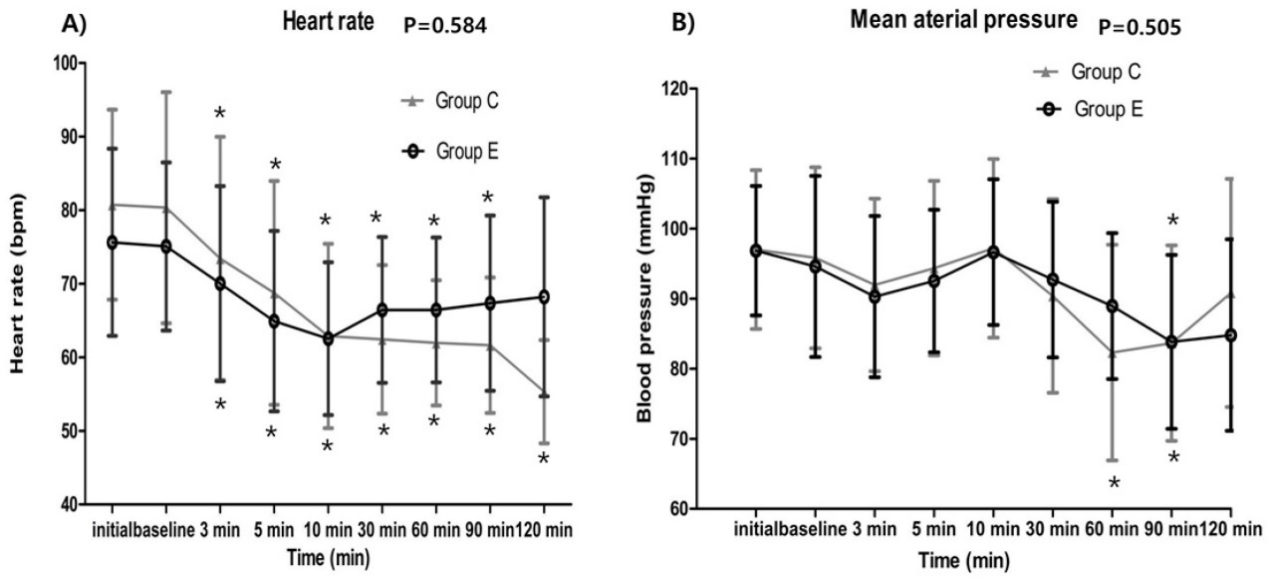
Hemodynamic data showing no significant intergroup differences between the control and ephedrine group: A) Heart rate (p = 0.584); B) Mean arterial pressure (p = 0.505). The x-axis represents the time point from preanesthetic point through 120 minutes. Preanesthetic, before performing spinal anesthesia; baseline, starting the loading dose of dexmedetomidine; ^*^p <0.05, compared with the corresponding baseline value.

The serially assessed HR (baseline p = 0.042, 3 min p = 0.222, 5 min p = 0.139, 10 min p = 0.869, 30 min p = 0.037, 60 min p = 0.022, 90 min p = 0.046, 120 min p = 0.053) and MAP (baseline p = 0.608, 3 min p = 0.453, 5 min p = 0.389, 10 min p = 0.798, 30 min p = 0.327, 60 min p = 0.020, 90 min p = 0.959, 120 min p = 0.439) did not exhibit any significant intergroup differences. In both groups, HR was significantly decreased at all-time points compared to their corresponding baseline value (all time points p < 0.001) without 120 min in the ephedrine group (p = 0.007). MAP was also decreased at 60 min, 90 min time points compared to their corresponding baseline value in control group (3 min p = 0.013, 5 min p = 0.335, 10 min p = 0.437, 30 min p = 0.019, 60 min p < 0.001^*^, 90 min p = 0.001^*^, 120 min p = 0.473) and decreased at 90 min time points in ephedrine group (3 min p = 0.032, 5 min p = 0.274, 10 min p = 0.292, 30 min p = 0.407, 60 min p = 0.021, 90 min p = 0.004^*^, 120 min p = 0.018) (Figure 2.).

## Discussion

In the current trial, we found that pre-emptive IM ephedrine injection was efficacious in terms of reducing the incidence of hypotension or bradycardia and the requirements of rescue ephedrine or atropine in patients who received spinal anesthesia combined with sedation via continuous infusion of dexmedetomidine.

Sedation using dexmedetomidine provides several potential benefits. First, dexmedetomidine is known to preserve spontaneous respiration and has the least potential to cause airway compromise among the commonly used sedatives 19. Second, sedation with dexmedetomidine elicits similar electroencephalography patterns to physiologic sleep 20, which may be beneficial in terms of postoperative delirium 21. Accordingly, patients can readily recover purposeful responsiveness, if necessary 22. Third, dexmedetomidine is unique among the sedatives in that it can also provide analgesia 19. Of particular relevance to spinal anesthesia, both intrathecal and intravenous administration of dexmedetomidine extend the duration of sensory and motor blockades, deepen sedation, delay the time required for postoperative rescue analgesics, and reduce consumption of morphine during the postoperative 24 hours 18, 23. Indeed, cumulating evidence shows that dexmedetomidine is superior in terms of satisfaction for both patient and interventionists compared with midazolam 16, 24-26. In addition, patients sedated with dexmedetomidine exhibited lower pain values and lower analgesic requirements postoperatively 26, 27.

However, being an alpha-2 agonist, dexmedetomidine use can result in bradycardia and hypotension caused by central sympatholytic effects 28. In theory, these sympatholytic features of dexmedetomidine could impose an incremental risk of hemodynamic perturbations by spinal anesthesia-induced sympathetic blockades when used in combination. In the literature, combined use of spinal anesthesia with dexmedetomidine increased the incidence of hypotension (16% versus 10%) and bradycardia (30% versus 0%) compared with spinal anesthesia alone in patients undergoing lower extremity surgery 18, mandating the need for an effective preventive measure to counteract the hemodynamic perturbations.

Ephedrine is a non-specific alpha and beta sympathetic agonist with a predominant beta effect that increases systolic blood pressure by increasing stroke volume and HR 13. Ephedrine is the most common rescue cardiotonic drug for treatment of hypotension accompanied by neuraxial anesthesia. Ephedrine is cost-effective and relatively safe to use in variety of patients receiving anesthetic care, even without an invasive arterial line. However, the pharmacokinetic properties of ephedrine do not allow continuous intravenous infusion, and it is mostly administered as intermittent intravenous bolus that results in peaks and troughs of plasma drug concentration. Moreover, considering that the potential ischemic harm conveyed by reduced MAP to major organs is dependent on the cumulative time spent below that low MAP 29, proactive treatments to maintain stable MAP and cardiac output instead of reactive treatments should be more encouraged. For that purpose, use of IM ephedrine has long been considered an efficacious and cost-effective method as it can provide continuous and sustained release of the drug 30. On the other hand, IM injection is usually discouraged due to the unpredictable onset or offset. Yet, previous studies have depicted a relatively reliable onset time of 15 to 20 minutes with its effects not lasting longer than 2 hours 15. Previous studies addressing the efficacy of pre-emptive IM ephedrine in spinal anesthesia showed promising results regarding the preventive effect of hemodynamic perturbations without untoward hypertensive or tachycardia side-effects 15, 31, including those undergoing Caesarean section requiring a high level of spinal block 32, 33. Evidence is lacking regarding the efficacy of pre-emptive IM ephedrine treatment in patients who received spinal anesthesia in combination with sedation using dexmedetomidine. In the present study, we showed that ephedrine 20 mg IM injection reduced hemodynamic perturbations in spinal anesthesia with dexmedetomidine (55.9% versus 26.3%, p = 0.001). It is meaningful to obtain hemodynamic stability through IM injection that maintains a constant concentration without reaching the peak. In addition, IM ephedrine reduced the use of rescue ephedrine and atropine as secondary endpoints. This result can be interpreted as reducing the severity of hypotension or bradycardia that may be translated into improved outcome, which merits a further study in that regard.

The limitations of the current study are as follows. The dose of ephedrine was not determined according to body mass. The 20 mg IM ephedrine injection in all patients had not been dosed in a tailored fashion. Second, the mean block height of spinal anesthesia was T10. In the literature, the odds ratio of hypotension increased more than 3 times when the level was T5 or higher 9. Therefore, if the target level had been higher, the preventive effect of ephedrine would have been better exhibited but would require an ephedrine dose higher than 20 mg, which was beyond the scope of the current study.

In conclusion, a single pre-emptive ephedrine 20 mg IM injection may be a safe and efficacious option to prevent sympathetic blockade-induced hemodynamic perturbations manifested as hypotension or bradycardia in patients who received spinal anesthesia and sedation using dexmedetomidine.

## Acknowlegements

J.H.P. and H.K.L. contributed to the conception and design, and revised manuscript; J.H.P. and J.K.S. drafted the manuscript; J.H.P. and H.H contributed to data acquisition and interpretation; J.H.P. and J.K.S. contributed to analyzing the clinical data from the data base.

## Figures and Tables

**Table 1:** Patients' characteristics

	Control (n = 59)	Ephedrine (n = 57)	P-value
Age (yrs)	53.0 ± 16.7	54.1 ± 18.2	0.734
Sex (M/F)	30/29	24/33	0.347
Height (cm)	161.2 ± 11.1	162.3 ± 9.9	0.600
Body surface area (m^2^)	1.72 ± 0.20	1.72 ± 0.19	0.995
Hypertension	25 (42.4)	19 (33.3)	0.318
Diabetes mellitus	17 (28.8)	13 (22.8)	0.462
Chronic kidney disease	2 (3.4)	2 (3.5)	0.972
Cerebrovascular accident	4 (6.8)	1 (1.8)	0.185
Medications			
Beta-blocker	8 (13.6)	7 (12.3)	0.838
ACEi	0 (0.0)	2 (3.5)	0.148
ARB	20 (33.9)	13 (22.8)	0.188
CCB	13 (22.0)	9 (15.8)	0.393
Diuretics	5 (8.5)	9 (15.8)	0.229
ASA class			0.141
I	17 (28.8)	25 (43.9)	
II	35 (59.3)	26 (45.6)	
III	7 (11.9)	6 (10.5)	
Block height	T 9.6 ± 1.7	T 9.7 ± 1.6	0.853
Surgical time	75.08 ± 31.91	70.35 ± 33.96	0.441

Data are displayed in mean ± SD or n (%). P value, intergroup comparison between the control and ephedrine group; ACEi, angiotensin converting enzyme inhibitor; ARB, angiotensin receptor blocker; CCB, calcium channel blocker; ASA, American Society of Anesthesiologists; T, thoracic dermatome.

**Table 2:** Primary and secondary endpoints.

	Control (n=59)	Ephedrine (n=57)	P-value
**Primary endpoint**			
**Overall**	**33 (55.9)**	**15 (26.3)**	0.001*
Bradycardia	20 (33.9)	8 (14.0)	0.013*
Hypotension	16 (27.1)	9 (15.8)	0.140
**Secondary endpoints**			
Atropine (mg)	0.28 ± 0.41	0.09 ± 0.21	0.001*
Ephedrine (mg)	2.03 ± 3.25	1.04 ± 2.89	0.007*
Adverse events			
Intra OP hypertension	7 (11.9)	4 (7.0)	0.375
Intra OP tachycardia	2 (3.4)	1 (1.8)	0.581
Intra OP anti-emetics	2 (3.4)	3 (5.3)	0.621
Post OP anti-emetics	41 (69.5)	36 (63.2)	0.472

Data are displayed in n (%) or mean ± SD, ^*^P <0.05, intergroup comparisons between the control and ephedrine group; OP, operation.

## References

[1] De Andres J, Valia JC, Gil A, Bolinches R (1995). Predictors of patient satisfaction with regional anesthesia. Reg Anesth.

[2] DeBenedittis G, Cigada M, Bianchi A, Signorini MG, Cerutti S (1994). Autonomic changes during hypnosis: a heart rate variability power spectrum analysis as a marker of sympatho-vagal balance. Int J Clin Exp Hypn.

[3] Barends CR, Absalom A, van Minnen B, Vissink A, Visser A (2017). Dexmedetomidine versus midazolam in procedural sedation. a systematic review of efficacy and safety. PLoS One.

[4] Carollo DS, Nossaman BD, Ramadhyani U (2008). Dexmedetomidine: a review of clinical applications. Curr Opin Anaesthesiol.

[5] Abdallah FW, Abrishami A, Brull R (2013). The facilitatory effects of intravenous dexmedetomidine on the duration of spinal anesthesia: a systematic review and meta-analysis. Anesth Analg.

[6] Niu XY, Ding XB, Guo T, Chen MH, Fu SK, Li Q (2013). Effects of intravenous and intrathecal dexmedetomidine in spinal anesthesia: a meta-analysis. CNS Neurosci Ther.

[7] Kim D, Jeong JS, Park H, Sung K-S, Choi SJ, Gwak MS (2019). Postoperative pain control after the use of dexmedetomidine and propofol to sedate patients undergoing ankle surgery under spinal anesthesia: a randomized controlled trial</p>. J Pain Res.

[8] Arain SR, Ebert TJ (2002). The efficacy, side effects, and recovery characteristics of dexmedetomidine versus propofol when used for intraoperative sedation. Anesth Analg.

[9] Carpenter RL, Caplan RA, Brown DL, Stephenson C, Wu R (1992). Incidence and risk factors for side effects of spinal anesthesia. Anesthesiology.

[10] Jo YY, Lee D, Jung WS, Cho NR, Kwak HJ (2016). Comparison of intravenous dexmedetomidine and midazolam for bispectral index-guided sedation during spinal anesthesia. Med Sci Monit.

[11] Song J, Kim WM, Lee SH, Yoon MH (2013). Dexmedetomidine for sedation of patients undergoing elective surgery under regional anesthesia. Korean J Anesthesiol.

[12] Hong JY, Kim WO, Yoon Y, Choi Y, Kim SH, Kil HK (2012). Effects of intravenous dexmedetomidine on low-dose bupivacaine spinal anaesthesia in elderly patients. Acta Anaesthesiol Scand.

[13] Critchley LA, Stuart JC, Conway F, Short TG (1995). Hypotension during subarachnoid anaesthesia: haemodynamic effects of ephedrine. Br J Anaesth.

[14] Goertz AW, Hubner C, Seefelder C, Seeling W, Lindner KH, Rockemann MG (1994). The effect of ephedrine bolus administration on left ventricular loading and systolic performance during high thoracic epidural anesthesia combined with general anesthesia. Anesth Analg.

[15] Sternlo JE, Rettrup A, Sandin R (1995). Prophylactic i.m. ephedrine in bupivacaine spinal anaesthesia. Br J Anaesth.

[16] Park JH, Soh S, Kwak YL, Kim B, Choi S, Shim JK (2019). Anesthetic efficacy of dexmedetomidine versus midazolam when combined with remifentanil for percutaneous transluminal angioplasty in patients with peripheral artery disease. J Clin Med.

[17] Consales G, Chelazzi C, Rinaldi S, De Gaudio AR (2006). Bispectral index compared to Ramsay score for sedation monitoring in intensive care units. Minerva Anestesiol.

[18] Elcicek K, Tekin M, Kati I (2010). The effects of intravenous dexmedetomidine on spinal hyperbaric ropivacaine anesthesia. J Anesth.

[19] Ebert TJ, Hall JE, Barney JA, Uhrich TD, Colinco MD (2000). The effects of increasing plasma concentrations of dexmedetomidine in humans. Anesthesiology.

[20] Akeju O, Brown EN (2017). Neural oscillations demonstrate that general anesthesia and sedative states are neurophysiologically distinct from sleep. Curr Opin Neurobiol.

[21] Duan X, Coburn M, Rossaint R, Sanders RD, Waesberghe JV, Kowark A (2018). Efficacy of perioperative dexmedetomidine on postoperative delirium: systematic review and meta-analysis with trial sequential analysis of randomised controlled trials. Br J Anaesth.

[22] Guldenmund P, Vanhaudenhuyse A, Sanders RD, Sleigh J, Bruno MA, Demertzi A (2017). Brain functional connectivity differentiates dexmedetomidine from propofol and natural sleep. Br J Anaesth.

[23] Zhang H, Li M, Zhang SY, Fu M, Zhang SY (2016). Intravenous dexmedetomidine promotes spinal bupivacaine anesthesia and postoperative analgesia in lower limb surgery: a double-blind, randomized clinical CONSORT study. Medicine.

[24] Alhashemi JA (2006). Dexmedetomidine vs midazolam for monitored anaesthesia care during cataract surgery. Br J Anaesth.

[25] Apan A, Doganci N, Ergan A, Buyukkocak U (2009). Bispectral index-guided intraoperative sedation with dexmedetomidine and midazolam infusion in outpatient cataract surgery. Minerva Anestesiol.

[26] Barends CRM, Absalom A, Van Minnen B, Vissink A, Visser A (2017). Dexmedetomidine versus midazolam in procedural sedation. a systematic review of efficacy and safety. PLOS ONE.

[27] Gurbet A, Basagan-Mogol E, Turker G, Ugun F, Kaya FN, Ozcan B (2006). Intraoperative infusion of dexmedetomidine reduces perioperative analgesic requirements. Can J Anaesth.

[28] Bloor BC, Ward DS, Belleville JP, Maze M (1992). Effects of intravenous dexmedetomidine in humans. II. Hemodynamic changes. Anesthesiology.

[29] Walsh M, Devereaux PJ, Garg AX, Kurz A, Turan A, Rodseth RN (2013). Relationship between intraoperative mean arterial pressure and clinical outcomes after noncardiac surgery: toward an empirical definition of hypotension. Anesthesiology.

[30] Jin JF, Zhu LL, Chen M, Xu HM, Wang HF, Feng XQ (2015). The optimal choice of medication administration route regarding intravenous, intramuscular, and subcutaneous injection. Patient Prefer Adherence.

[31] Hemmingsen C, Poulsen JA, Risbo A (1989). Prophylactic ephedrine during spinal anaesthesia: double-blind study in patients in ASA groups I-III. Br J Anaesth.

[32] Ayorinde BT, Buczkowski P, Brown J, Shah J, Buggy DJ (2001). Evaluation of pre-emptive intramuscular phenylephrine and ephedrine for reduction of spinal anaesthesia-induced hypotension during Caesarean section. Br J Anaesth.

[33] Cleary-Goldman J, Negron M, Scott J, Downing RA, Camann W, Simpson L (2005). Prophylactic ephedrine and combined spinal epidural: maternal blood pressure and fetal heart rate patterns. Obstet Gynecol.

